# Advanced stage of breast cancer hoist alkaline phosphatase activity: risk factor for females in India

**DOI:** 10.1007/s13205-012-0113-1

**Published:** 2013-01-06

**Authors:** A. K. Singh, A. Pandey, M. Tewari, R. Kumar, A. Sharma, K. A. Singh, H. P. Pandey, H. S. Shukla

**Affiliations:** 1 Department of Surgical Oncology, Institute of Medical Sciences, Banaras Hindu University, Varanasi, 221005 India; 2 Amity Institute of Biotechnology, Amity University, Lucknow, UP India; 3 Department of Pharmaceutics, I.I.T. Banaras Hindu University, Varanasi, India; 4 Department of Biochemistry, Faculty of Science, Banaras Hindu University, Varanasi, India

**Keywords:** Breast cancer, Alkaline phosphatase, Metastasis, Biochemical parameter, Regression

## Abstract

Breast cancer is the most common neoplasm affecting women in the western world with an average frequency of 1 in 11, developing the malignancy and it is second most common cancer in India. Variations in serum levels of biochemical parameters especially alkaline phosphatase (ALP) changes may be of great help in diagnosis of breast carcinoma. Serum ALP activity was assayed in 388 histopathologically proven breast cancer patients using spectrophotometric methods and monitored association with cancer stages. Breast cancer is a female-biased disease and our study was conducted in a group of female patients with mean age of 48.67 ± 8.32 years. A significant increase in levels of ALP (809.65 ± 145.97 IU/L) was observed in stage IV of the disease. The logistic regression study gave a significant result (*P* < 0.001) when we compared the group of ALP level (>500 IU/L) with metastatic presentation. The present study besides being cost effective suggested the usefulness of ALP in differentiating breast cancer stages and metastasis.

## Introduction

Alkaline phosphatases [ALPs; orthophosphoric monoester phosphohydrolase (alkaline optimum) EC 3.1.3.1] are a group of phosphatidylinositol-anchored membrane proteins with wide substrate specificity (Fishman 1987; Harris 1990). It comprises a group of enzymes that catalyze the hydrolysis of phosphate esters in an alkaline environment, generating an organic radical and inorganic phosphate (Reichling and Kaplan 1988a, b). Like other enzymes, ALP has many isoenzymes. In healthy adults, this enzyme is mainly derived from the liver, bones and in lesser amounts from intestines, placenta, kidneys and leukocytes (Friedman et al. 1996). An increase in serum ALP levels is frequently associated with a variety of diseases such as extrahepatic bile obstruction, intrahepatic cholestasis, infiltrative liver disease, hepatitis, cancer, etc. However, the elevation of ALP less than three times the normal level is generally considered to be non-specific and insufficient to provide a definite diagnosis (McIntyre and Rosalki 1991).

Markedly elevated serum ALP, hyperalkalinephosphatasemia is predominantly seen with more specific disorders, including malignant biliary obstruction, primary biliary cirrhosis, primary sclerosing cholangitis, hepatic lymphoma, breast cancer and sarcoidosis (Neuschhwander-Terti 1995). On the other hand, according to a recent study (Maldonado 1998), sepsis and malignant obstructions are identified as common causes of hyperalkalinephosphatasemia, whereas diffuse liver metastases as well as a number of benign disorders are relatively less common causes of hyperalkalinephosphatasemia.

Cancer is cured potentially if it is detected early when the tumor is small enough and may be completely removed surgically. However, most cancers do not produce any symptoms until the tumors are either too large to be removed surgically or cancerous cells have already spread to other tissues as generally observed in metastasis (Mishra et al. 2004). Hence, there is a need to detect cancer at an early stage. Biochemical parameter like ALP will help to monitor the disease.

In order to determine the diseases associated with markedly elevated serum ALP levels among breast cancer hospitalized patients, a review was made of medical records of individuals in whom an ALP level was two- to threefold greater than normal ALP level in Department of Surgical Oncology, Sir Sundarlal Hospital, Banaras Hindu University, Varanasi (UP), INDIA.

## Methods

### Study population and clinical evaluation of patients

This consecutive cluster study included all patients who attended the OPD for cancer diagnosis and treatment at the Surgical Oncology Department, Sir Sundarlal Hospital, Banaras Hindu University. Exclusion criteria included patients <18 years of age, who received prior treatment in the form of chemotherapy/radiotherapy/surgery and patients who were mentally incapable of giving their own consent.

If the patient met the appropriate criteria, we visited the patient before surgery to explain the study and asked for patient’s participation. On receiving consent from the patient, we conducted a 30-min interview with the patient. Data involving presentation, diagnosis and staging were collected from office and hospital charts, and a face-to-face interview with the patient was done. The interview was mainly based on a predesigned questionnaire that included several questions about age, residence, health care and utilization.

### Sample and data collection

Five milliliters of whole blood samples were collected from each subject into plain tubes with utmost care to avoid pre-analytical errors. Serum was extracted for analysis of ALP. The activities of ALP were determined using the enzymatic method (King 1946).

The normal value of ALP is 20–140 IU/L. Normal value ranges may vary slightly among different laboratories.

A retrospective case review was made on hospitalized patients who had an ALP level ≥500 IU/L. The review of the patient’s medical records during the period of study identified 388 cases with a conclusive diagnosis for further analysis.

### Statistical analysis

Association of ALP level was compared among the different stages of breast cancer. Two-tailed tests were used at all times, and statistical significance was set a priori at *P* < 0.05. Statistical analyses were performed with SPSS for Windows 16.0. All univariate analyses used ANOVA and Chi-square tests. Logistic regression was performed to evaluate ALP level associated with the metastasis.

## Result and discussion

Breast cancer is leading cause of death all over the world, but till date there is a very little knowledge about the biological markers that help to diagnose the disease. Biochemical parameter like ALP evaluation is an inexpensive and potential marker for early detection of cancer that helps to diagnose the people of developing countries. Most data indicate that the elevation of serum ALP occurs because of the accelerated de novo synthesis of the enzyme and subsequent regurgitation into the serum (Reichling and Kaplan 1988a, b; Friedman et al. 1996). A number of diseases are related to the elevation of serum ALP levels.

The breast cancer is a female-biased disease; the frequency of the disease in male patient is very low in comparison with female. It is evident from the present study as in Table 1 that the breast cancer arises in a female before the age of 50 years (48.67 ± 8.32 years), whereas in a male, the mean age of onset is 54.29 years. It suggests that this disease is hormone biased. Younger age was found to be at high risk (Somdatta and Baridalyne 2008) while another case–control study of Rao et al. (1994) conducted in India found that the mean age of breast cancer patients was 46.2 years.

**Table 1:** Age and sex distribution

Breast cancer patient (*N* = 388)	Mean age
Male (*N* = 7)	54.29 ± 7.25
Female (*N* = 381)	48.67 ± 8.32

**Fig. 1:**
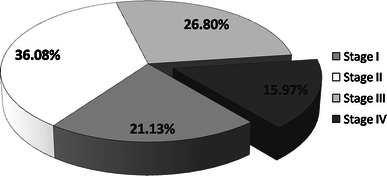
Distribution of cancer patients stage group in the study cohort (*N* = 388)

The distribution of patients according to various stages of cancer is indicated in Fig. 1. The result of the current study showed a significant increased level of ALP with the advancement of the disease. Table 2 shows the mean ALP levels at stage I, II, III and IV as 161.15 ± 110.06, 299.58 ± 111.60, 517.69 ± 110.28 and 809.65 ± 145.97, respectively.

**Table 2:** Distribution of alkaline phosphatase level with disease stage

Breast cancer stage	Alkaline phosphatase (IU/L)
Stage I	161.15 ± 110.06
Stage II	299.58 ± 111.60
Stage III	517.69 ± 110.28
Stage IV	809.65 ± 145.97

Table 3 shows the comparison between the two groups of ALP level that is less than 500 IU/L and more than 500 IU/L with cancer stage. Out of 62 cancer patients of stage IV, the ALP was more than 500 IU/L in 61 patients.

**Table 3:** Association of disease stage with alkaline phosphatase level

Parameter	Disease stage				*P* value
	I	II	III	IV	
Alkaline phosphatase					<0.001
<500 IU/L	76	134	39	1	
>500 IU/L	6	6	65	61	

ALP has consistently been shown to predict bone metastases, and to some extent liver metastases, as expected on the basis of its biological activity. While some studies have reported fairly high sensitivity of ALP for bone and overall metastases detection, these studies included the use of specific isoenzymes in addition to total ALP values [Cancer Study Group (IBCSG) 1995]. The result of the present study showed higher serum ALP levels in the advanced stage of breast cancer patients (Table 4). In present study, logistic regression showed a high significant result and explored >500 IU/L ALP level as strongly associated with metastasis.

**Table 4:** Association of alkaline phosphatase with metastasis of disease

Variable	Odds ratio	95 % confidence interval (CI)		*P* value
		Lower	Upper	
Alkaline phosphatase level				
<500 IU/L	1.0	–	–	–
>500 IU/L	197.260	26.89	1446.56	<0.001

Similarly Vanhoof et al. (1992) and Stieber et al. (1992) did not find any significant difference in ALP levels in non-metastatic breast cancer, despite the fact that some others have also revealed a significant rise in ALP in metastasis (Ramaswamy et al. 2000; Lamerz et al. 1993) suggesting involvement of bone and liver. A recent study of Mishra et al. (2004) also found persistent rise of ALPs level in metastasis (Ehrmeyer et al. 1978; Larson et al. 1976).

The increase in ALP level as noticed in our study also indicates that the disease had metastasized either to bone or liver.

## Conclusion

Women with breast cancer have ALP activities generally higher than normal healthy women. The progressive increase in the serum ALP activities with breast cancer is an indication of metastasis. The measurement of this parameter may be an useful diagnostic tool in monitoring the disease, its progression and treatment in areas where the facilities for sophisticated studies are not readily available.
